# Single-Electron-Transfer-Mediated Carbonylation Reactions

**DOI:** 10.1021/acs.accounts.5c00039

**Published:** 2025-03-05

**Authors:** Le-Cheng Wang, Xiao-Feng Wu

**Affiliations:** † Dalian National Laboratory for Clean Energy, Dalian Institute of Chemical Physics, Chinese Academy of Sciences, Dalian 116023, China; ‡ Leibniz-Institut für Katalyse e.V., Rostock 18059, Germany

## Abstract

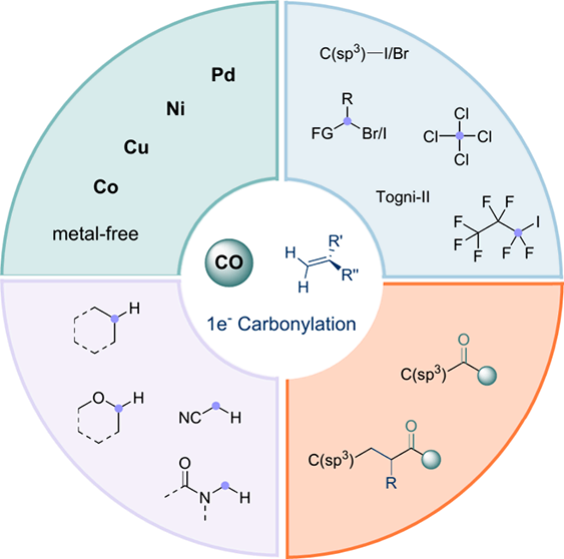

Transition-metal-catalyzed carbonylation coupling methods have been accepted as an essential tool for producing carbonylated products over the past few decades. Despite its long-standing history and widespread industrial applications, several challenges remain in carbonylation chemistry. These include reliance on precious metal catalysts, the need of high-energy radiation, difficulties in carbonylation of unactivated chemical bonds, etc. As an alternative to classic two-electron transfer process, single-electron-transfer (SET)-mediated carbonylation has emerged as a powerful tool to achieve elusive carbonylation transformations. Over the past few years, carbonylation of commonly available functional handles, such as alkenes and alkyl halides, via the single-electron pathway has emerged as a valuable area of research.

Our team has been dedicated to developing new carbonylation reactions using bulk chemicals to construct high-value carbonylated products. These reactions have broad synthetic and industrial applications, motivating us to explore SET-mediated carbonylation transformations for two key classes of bulk chemicals: alkanes and alkyl halides. Specifically, our work has centered on two main approaches: (1) Single-electron reduction of C(sp^3^)–X bonds: this strategy leverages single-electron reduction to activate C(sp^3^)–X bonds, promoting the formation of carbon radicals, which in turn promotes subsequent addition to metals or CO. However, a significant challenge lies in the highly negative reduction potential of certain substrates [E_red_ < −2 V compared to the saturated calomel electrode (SCE) for unactivated alkyl iodides]. Despite these challenges, the intrinsic reducibility of CO and the reactivity of various carbonyl-metal intermediates facilitate smooth reaction progress. (2) Single-electron oxidative of C(sp^3^)–H bonds: this strategy emphasizes efficiency, high atomic utilization, and minimal waste by bypassing traditional preactivation methods. Using 3d metal catalysts, we have successfully performed aminocarbonylation and alkoxycarbonylation on a wide range of C(sp^3^)–H bonds (such as those in aliphatic alkanes, ethers, amines, etc.). The above two approaches also enabled radical relay carbonylation of alkenes, allowing precise control over reaction intermediates and pathways. Such control improves both reaction efficiency and selectivity. These advancements have enabled transition metal or photoredox catalysis to facilitate radical relay carbonylation of unactivated alkenes, resulting in transformations such as oxyalkylative carbonylation, aminoalkylative carbonylation, fluoroalkylative carbonylation, double carbonylation, and rearrangement carbonylation.

SET-mediated carbonylation significantly enhances the sustainability and scalability of the carbonylation process by reducing reliance on precious metal catalysts and enabling milder reaction conditions. Additionally, by carefully controlling reaction intermediates, we have fine-tuned the process to produce a wide range of carbonylation products with high selectivity. This flexibility expands the applications of carbonylation in synthetic chemistry and industrial processes. Finally, we place particular emphasis on the application of carbonylation reactions in drug discovery, where they serve as powerful functional handles for the late-stage modification of bioactive molecules. The broad applicability of SET-mediated carbonylation methods to various chemical bonds significantly enriches the toolbox for drug synthesis, enabling the efficient functionalization of complex molecules. This versatile approach has the potential to accelerate the discovery of novel therapeutic agents, making it a critical tool in modern medicinal chemistry.

## Key References

- ZhaoF.; AiH.-J.; WuX.-F.Copper-Catalyzed Substrate-Controlled Carbonylative Synthesis of α-Keto Amides and Amides from Alkyl Halides. Angew. Chem., Int. Ed.2022, 61, e20220006210.1002/anie.20220006235175679.^1^ This work first achieved copper-catalyzed controlled double- and monocarbonylation of alkyl halides with amines.
- WangY.; YangH.; ZhengY.; HuM.; ZhuJ.; BaoZ.-P.; ZhaoY.; WuX.-F.Carbon Monoxide Enabling Synergistic Carbonylation and (Hetero)aryl Migration. Nat. Catal.2024, 7, 1065–107510.1038/s41929-024-01204-6.^2^ This work realized the synergistic carbonylation and (hetero)aryl migration, and its key mechanistic innovation lies in the utilization of CO as a bridge to allow for a necessary five-membered transition state for aryl migration.
- LiY.; DongK.; ZhuF.; WangZ.; WuX.-F.Copper-Catalyzed Carbonylative Coupling of Cycloalkanes and Amides. Angew. Chem., Int. Ed.2016, 55, 7227–723010.1002/anie.20160323527167881.^3^ The copper-catalyzed C–H carbonylation of alkanes by direct hydrogen atom transfer using CO as the economic and abundant C1 source was first developed.
- WangL.-C.; YuanY.; ZhangY.; WuX.-F.Cobalt-Catalyzed Aminoalkylative Carbonylation of Alkenes Toward Direct Synthesis of γ-Amino Acid Derivatives and Peptides. Nat. Commun.2023, 14, 7439–744810.1038/s41467-023-43306-y37978196 PMC10656502.^4^ γ-Amino acid and its derivatives are well-appreciated, yet efficient and modular syntheses are scarce. This work described cobalt-catalyzed aminoalkylative carbonylation of alkenes for the efficient synthesis of γ-amino acid and its derivatives.

## 1 Introduction

The controlled incorporation of carbon monoxide (CO) into organic molecules, known as carbonylation, is a highly valuable chemical transformation with a significant role in the chemical industry and organic synthesis.^5^ This type of reaction effectively connects different functional groups at both ends of the carbonyl group, enabling straightforward extension of the parent compound’s carbon chain by introducing one or more CO molecules. CO is an abundant carbon-based molecule that can be derived from diverse resources, including naphtha, coal, natural gas, biomass, and so on.^6^ Drawing inspiration from transition metal catalysts, numerous catalytic carbonylation reactions have been developed, many of which are widely employed in industrial applications, such as the Fischer–Tropsch synthesis and the Monsanto-Cativa acetic acid process.^7^ Not surprisingly, advances in CO activation, catalytic systems, and reaction models are critical for improving these transformations’ selectivity, efficiency, and sustainability, contributing to the development of resource-efficient chemical processes for the future.

**Figure 1:**
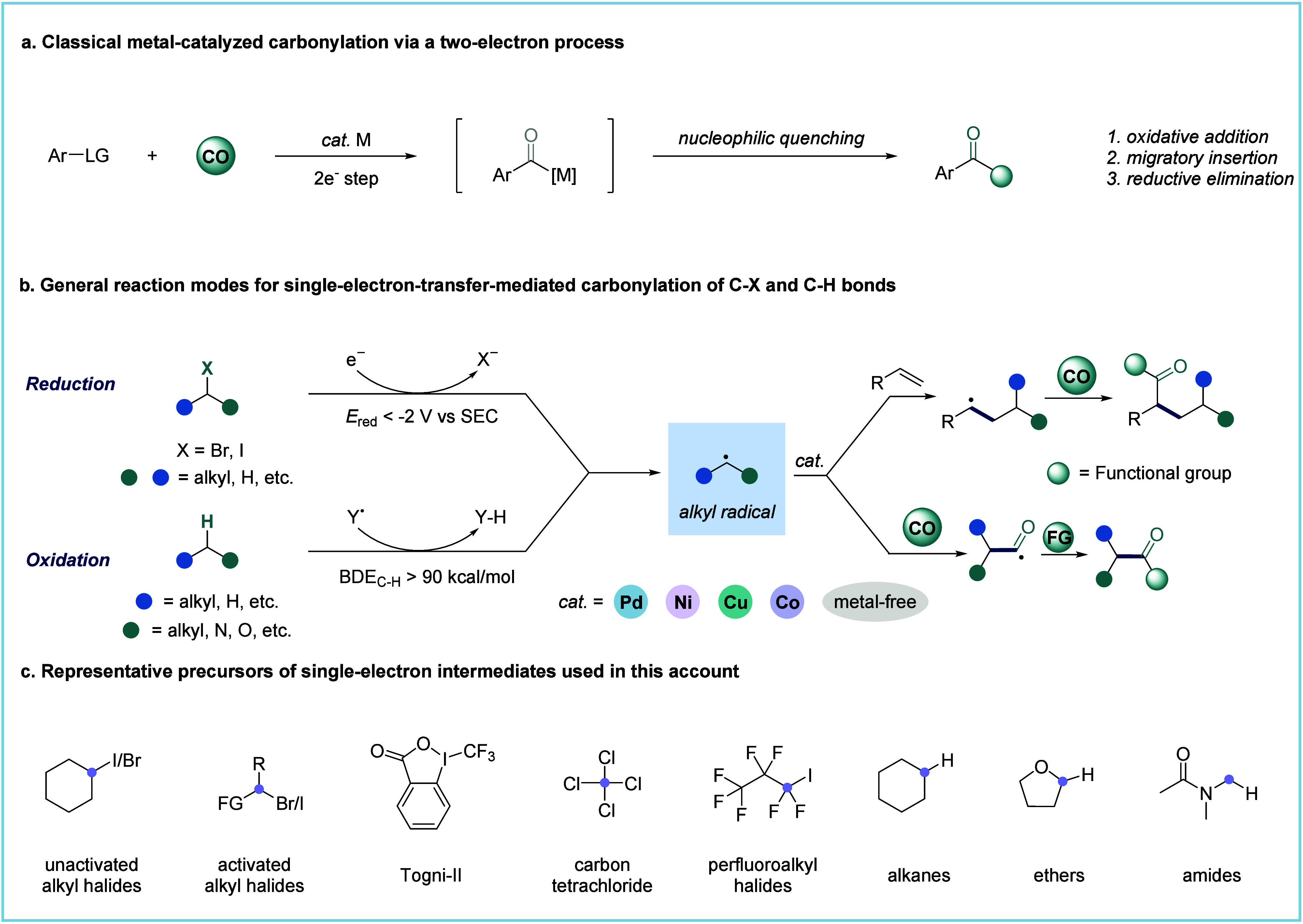
Development of various SET-mediated carbonylative transformations from C–X or C–H bonds.

Traditional transition metal-catalyzed carbonylation reactions typically rely on precious metal catalysts and proceed via the classic two-electron oxidative addition/CO migratory insertion/reductive elimination cycle (Figure 1A).^8^ The strong π-acidic nature of CO enables its coordination with transition metals, facilitating the feedback electron transfer from the metal center to the antibonding orbitals of CO. This interaction reduces the electron density at the metal center, which weakens its nucleophilicity and makes oxidative addition more challenging.^9^ Consequently, two-electron-mediated carbonylation predominantly favors substrates with C(sp^2^) hybridization. In contrast, the activation of C(sp^3^) substrates via the oxidative addition is often less efficient due to slower reaction rates. Furthermore, the presence of unstable and open coordination sites frequently accelerates undesirable β-hydride elimination, posing additional challenges.^10^

In contrast, the single-electron-transfer (SET)-mediated carbonylation involves the transfer of a single electron to generate a reactive single-electron species. This process is typically driven by a radical mechanism, initiated by single-electron-transfer, rather than the traditional two-electron-transfer characteristic of most metal-catalyzed processes. In this context, the reactive single-electron species can be quenched through two pathways: (1) by carbon monoxide or (2) by metal catalysts. In the first pathway, a carbon-centered radical reacts with CO, forming an acyl radical that features a nucleophilic singly occupied molecular orbital (SOMO) and an electrophilic carbonyl π*. Here, the CO molecule acts as an electron-deficient species.^11^ Early established reaction class in this field was primarily atom transfer carbonylation, which relies on high-energy radiation or radical initiators to release reactivity.^12^ For the latter case, the generated carbon-centered radical undergoes a single-electron oxidation, transferring one electron to the metal center of the catalyst. This stabilizes the radical by forming a covalent bond with the metal, resulting in an alkyl-metal intermediate. Subsequent metal single-electron reduction of the metal further broadens the boundaries of these reactions, enabling the formation of diverse carbonylation products.^13^ We envision that carbonylation transformations could greatly benefit from adopting a single-electron transfer pathway in several key aspects: (1) the single-electron pathway bridges the gap between inert substrates and the thermodynamically unstable CO, facilitating reactivity with challenging substrates; (2) the high reactivity of single-electron intermediates reduces dependence on noble metal catalysts; (3) cost-effective and readily available 3d metals are better suited for single-electron transfer mechanisms compared to the 4d and 5d metals typically used in two-electron chemistry.

**Figure 2:**
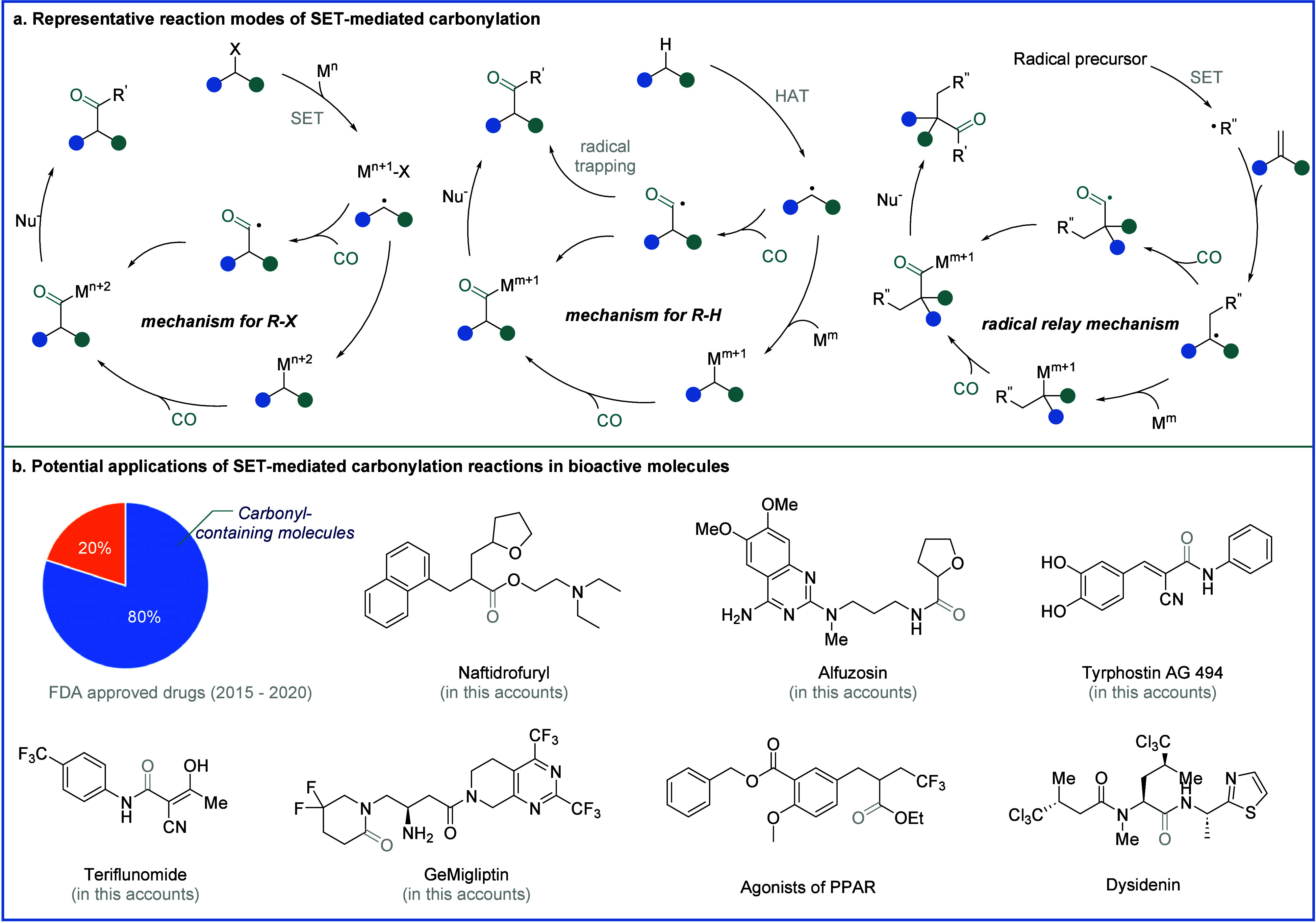
A. Representative reaction modes of single-electron-mediated carbonylation. B. Potential applications of SET-mediated carbonylation reactions in bioactive molecules.

Below, we present our laboratory’s research on SET-mediated carbonylation transformations. To expand the catalytic platforms for carbonylation reactions, we have developed two distinct catalytic strategies (Figure 1B, and more details mechanisms are explained in Figure 2A). (1) Single-electron reduction of C(sp^3^)–X bonds: in this approach, C(sp^3^)–X substrates undergo single-electron reduction with low valence metal, resulting in the formation of M^n+1^ and a C(sp^3^) radical, which subsequently bonds to the metal center. Additionally, charge transfer under photocatalytic redox conditions offers a promising solution to the challenges of sustainable carbonylation chemistry. This strategy focuses on designing novel SET-mediated carbonylation reactions for unactivated alkyl halides, intending to avoid harsh reaction conditions, such as high CO pressure or the use of noble and even metal catalysts. (2) Single-electron oxidation of C(sp^3^)–H bonds: this strategy efficiently activates C(sp^3^)–H substrates through hydrogen atom transfer (HAT), leading to the generation of carbon-centered radicals. However, the strong bond dissociation energies of C(sp^3^)–H present a challenge for single-electron oxidation, particularly under reductive CO atmospheres. In both of the above strategies, single-electron transfer offers a direct pathway for generating radical precursors, which in turn promote the formation of carbon radicals from alkenes and facilitate subsequent carbonylation reactions via a radical relay mechanism. A key challenge in these reactions is controlling the reaction dynamics to ensure precise assembly of the components and coordinating the reactivity of intermediates with other substrates to achieve high selectivity. Effective management of these dynamics is critical for advancing the scope and precision of SET-mediated carbonylation transformations. In this Account, we systematically discuss the latest reaction patterns achieved by our group in SET-mediated carbonylation reactions, with a focus on the utilization of CO in transformations involving alkyl halides, alkanes, and alkenes. Our findings highlight the potential of SET pathways to overcome traditional limitations in carbonylation chemistry, offering more efficient, selective, and sustainable solutions for carbonylation transformations. Finally, the SET-mediated carbonylation methods can significantly expand the toolkit for drug synthesis (Figure 2B).

## 2 Single-Electron-Reduction of C–X Bonds

### 2.1 Alkyl Halides

In recent decades, significant progress has been made in the field of carbon–halogen bonds carbonylation. These advancements are largely due to the pioneering work of Heck,^14^ Ryu,^15^ Alper,^16^ and more recent contributions by Arndtsen,^17^ Mankad,^18^ and our group.^19^ Many successful strategies for alkyl halide carbonylation, utilizing transition metal catalysis or photoinduced single-electron transfer processes, have been established. However, most of these methods have focused on alkyl iodides, and a broadly applicable method for carbonylation of organic halides with carbon monoxide under mild conditions remains elusive. Key challenges include poor functional group tolerance and competitive side reactions, such as over-reduction and dehalogenation hydrogenation, which are largely attributed to the highly negative reduction potential of halides. In this part, we highlight our recent advancements in alkyl halide carbonylation, including innovative reaction modes such as controlled double- and monocarbonylation, the use of inexpensive or metal-free systems, and the application of atmospheric pressure CO conditions. Additionally, we address strategies for activating reactive halides prone to dehalogenation hydrogenation.

**Figure 3:**
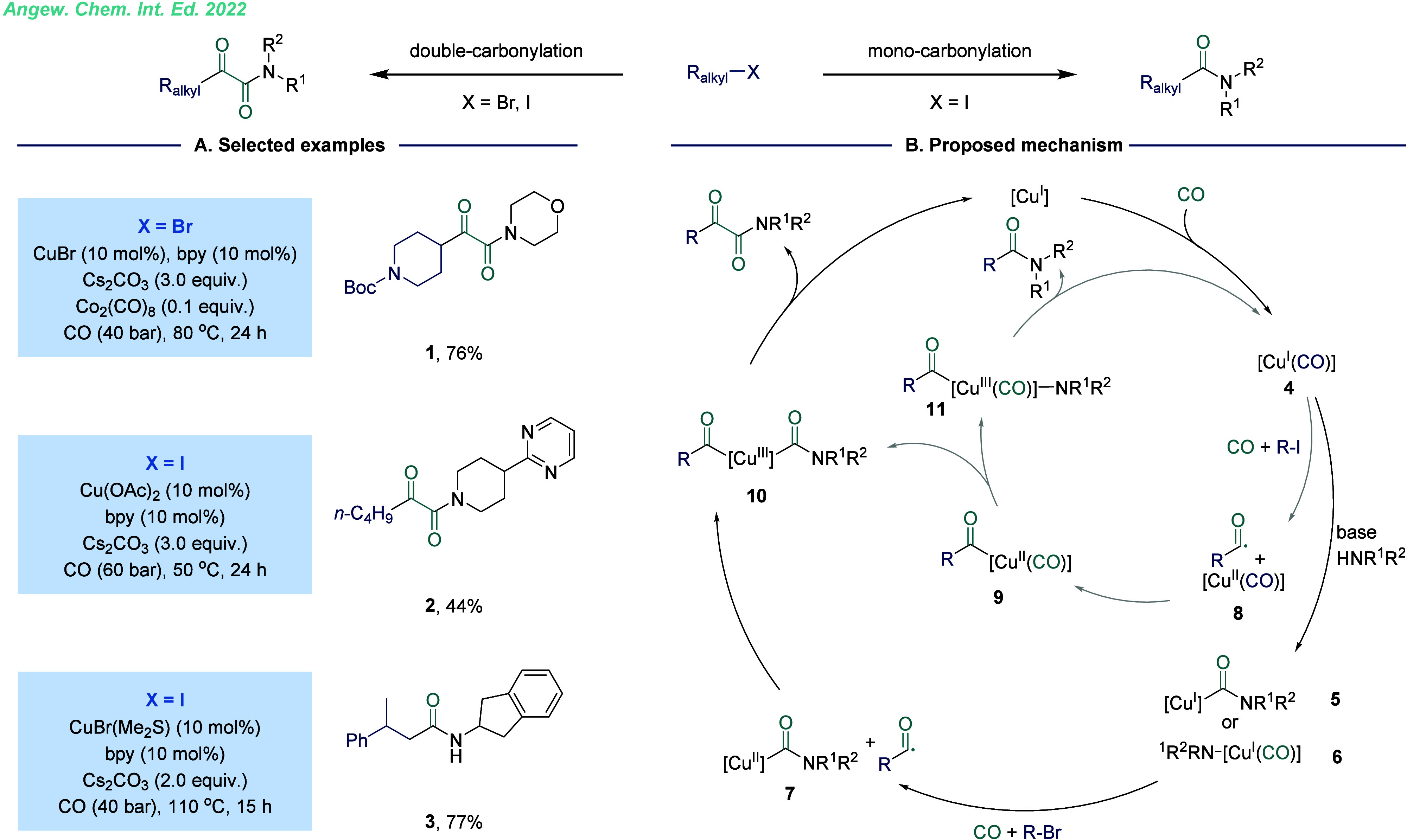
Copper-catalyzed substrate-controlled double- and monocarbonylation. A. Selected examples. B. Proposed mechanism.

The controlled introduction of one or more CO molecules into the same substrate has always been an attractive goal in the field of carbonylation. Despite the numerous catalytic methods for carbonylation reactions via ionic or radical pathways, a fundamental limitation of these methods is the difficulty in achieving high selectivity for mono- and double-carbonylation in a single reaction. In 2022, our group developed a copper-catalyzed, substrate-controlled carbonylative transformation to effectively convert alkyl halides to α-keto amides and amides (Figure 3).^1^ This method demonstrated exceptional selectivity for alkyl bromides, yielding a single double-carbonylated product, while alkyl iodides enabled controllable double- or monocarbonylation under different conditions. This approach features a broad substrate scope and has been successfully applied to the modification of complex natural products and pharmaceutical compounds. In this reaction, CO coordinated with the copper salt to form a (carbonyl)copper species **4**. Subsequently, amines engaged in nucleophilic attack on the coordinated CO, yielding a copper complex, followed by an intermolecular SET process, that generated an intermediate and an acyl radical. Alternatively, amines underwent an anionic ligand exchange with the (carbonyl)copper species to deliver an electron-rich amino copper(I) species. Next, the alkyl bromide was then activated through another SET process, leading to CO insertion and formation of the complex. The acyl radical reacted with this complex to form a copper(II) species, which then underwent a reductive elimination to release the desired product and regenerate the Cu(I) catalyst. For alkyl iodides, the activation may occur before the nucleophilic attack of amine on the coordinated CO due to the lower activation energy barrier of alkyl iodides compared to bromides. The corresponding intermediate then reacted with an acyl radical to form a key intermediate, which subsequently generated a copper(III) species. This species underwent an anionic exchange with amines in the presence of bases, forming an intermediate. Reductive elimination from this intermediate yielded the desired amide product. Additionally, the intermediate could undergo a nucleophilic attack by amines to form another intermediate, further diversifying the reaction pathway (Figure 3B).

**Figure 4:**
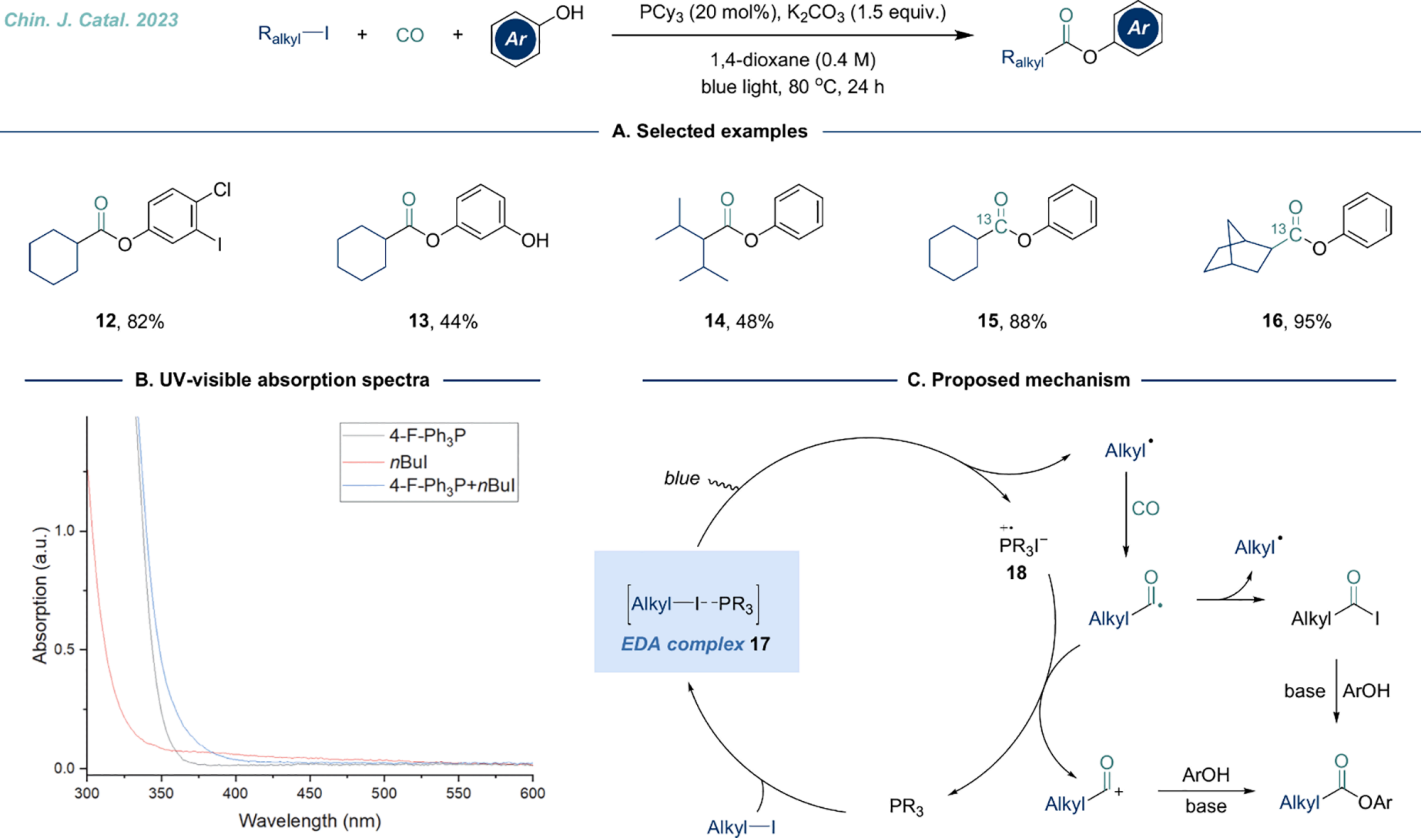
Phosphine-catalyzed photoinduced alkoxycarbonylation of alkyl iodides under 1 bar of CO. A. Selected examples. B. UV/visible absorption study. C. Proposed mechanism.

Significant progress has been made in the field of C–X carbonylation in recent decades, with most advances involving transition metal-catalyzed processes.^20^ However, developing a general method for carbonylation of organohalides under metal-free conditions remains challenging.^21^ In this case, alkyl radicals undergo rapid radical addition with CO to form acyl radicals. However, the process of generating acyl radicals is reversible, as the reverse decarbonylation step is thermodynamically favored. Consequently, high CO pressure (>40 bar) is typically required to drive the reaction forward. Inspired by our previous work on metal-catalyzed carbonylation of unactivated alkyl halides, we recently developed a phosphine-catalyzed, photoinduced alkoxycarbonylation of alkyl iodides via through electron donor–acceptor (EDA) photoactivation (Figure 4).^22^ This approach enabled carbonylative transformation of alkyl iodides under mild conditions, specifically at atmospheric pressure of CO (1 bar), without the need for transition metals. In this transformation, phosphines played a critical role in recognizing substrate types: tris(4-fluorophenyl)phosphine selectively activated primary alkyl iodides, while tricyclohexylphosphine was effective for secondary alkyl iodides. The method also demonstrates its utility in incorporating radioisotopes into metabolically stable sites, which is crucial for drug tracking and toxicity monitoring. A variety of carbon-13-labeled phenol esters were synthesized under 1 bar CO, minimizing isotope waste and highlighting the efficiency of this protocol. UV/visible absorption experiments confirmed the formation of an EDA complex, as evidenced by the red-shifted spectrum observed when 1-iodobutane was combined with tris(4-fluorophenyl)phosphine (Figure 4B). The key step in this reaction involved the formation of the EDA complex **17** between the alkyl iodide and phosphine. Upon blue light irradiation, the complex underwent activation, generating a phosphine radical ion pair **18** and an alkyl radical, which then participated in the carbonylation process (Figure 4C). Recently, our group successfully achieved a metal-free, exogenous catalyst-free aminocarbonylation of alkyl iodides under 1 bar CO.^23^ The reaction proceeds efficiently under light irradiation at room temperature through the combination of EDA and XAT strategies.

**Figure 5:**
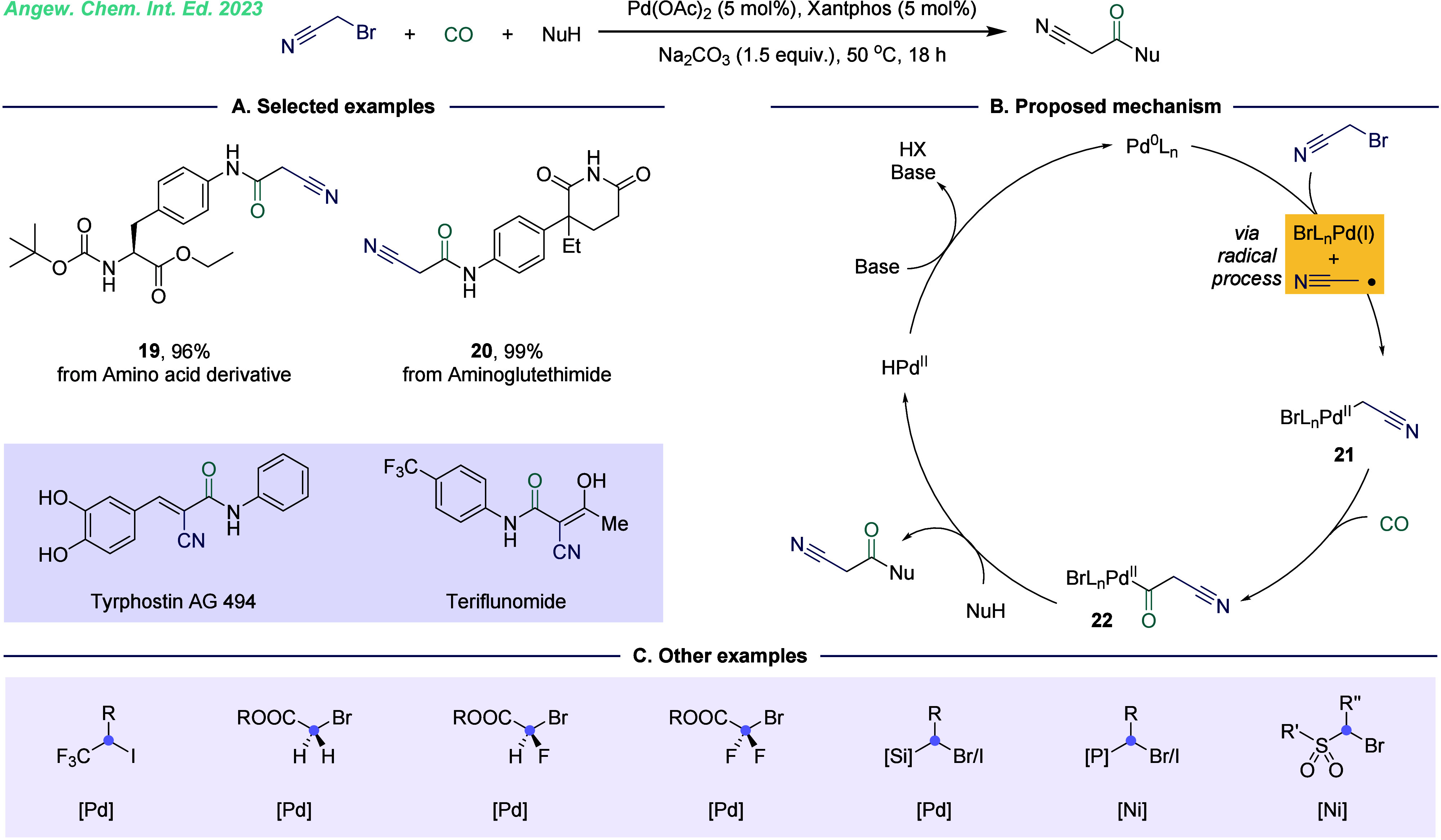
Palladium-catalyzed direct carbonylation of bromoacetonitrile. A. Selected examples. B. Proposed mechanism. C. Other examples for carbonylation of active halides.

The carbonylation of activated halides offers a simple and effective method for the synthesis of practical molecular framework. However, those processes often result in undesired nucleophilic substitution and dehydrohalogenation due to the high reactivity of the substrates. Additionally, performing two-electron chemistry with such highly active species is challenging. Recently, our group developed a facile metal-catalyzed single-electron transfer strategy involving metal-stabilized active carbon radicals (Figure 5).^24^ Using this approach, a wide variety of value 2-cyano-*N*-acetamide and 2-cyanoacetate molecules were synthesized with excellent yields and outstanding functional group compatibility. Furthermore, we successfully obtained 7 drug precursors in good yields, demonstrating a convenient and straightforward alternative synthetic route. The mechanistic studies revealed that bromoacetonitrile underwent a single-electron reduction with Pd(0) to generate a carbon-centered radical and Pd(I) intermediates. The C-radical was quickly captured by Pd(I) to form a Pd(II) intermediate **21**, which subsequently underwent CO migration insertion and reduction elimination to yield the target product (Figure 5B). Molecules containing functional groups such as phosphine, silicon, fluorine, and cyano groups offer significant advantages in various chemical reactions due to their unique electronic and steric properties. They not only have important applications in catalysis, synthesis, and pharmaceuticals, but also demonstrate significant synthetic value in materials science, electronics, and other fields. By rationally designing and modulating these functional groups, the functionality and application scope of the compounds can be greatly enhanced. Building on this foundation, we further explored SET-mediated carbonylation of active halides. The scope of this method now includes various substrates such as trifluoromethyl ester,^25^ monofluoroester, difluoroester,^26^ silicon,^27^ phosphine, and sulfonyl substituted α-halogenated hydrocarbons (Figure 5C).^28^ These advancements underscored the versatility and potential of SET-mediated carbonylation in synthesizing structurally diverse compounds.

### 2.2 Reduction-Induced Tandem Alkene Carbonylation

Alkenes are ubiquitous in natural products and functional materials, making them one of the most versatile functional groups in chemical synthesis. Additionally, they are essential raw materials in the chemical industry, driving the production of various fine chemicals, including, synthetic rubbers, fuels, and coatings, thereby forming a fundamental cornerstone of modern chemistry and industry.^29^ Multiple methods are available for the synthesis of alkenes, such as elimination reaction of (pseudo)halide,^30^ carbonyl olefination of ketones,^31^ and alkene metathesis.^32^ In comparison to the extensively studied organometallic catalysis of carbonylation, SET-mediated carbonylation of alkenes remains underexplored due to the inherent instabilities of single-electron species. For decades, radical relay strategies have been widely employed to utilize these alkenes in synthetic chemistry.^33^ We hypothesize that the single-electron platform could unlock previously unknown reactivity in alkene carbonylation reactions. Inspired by radical chemistry, we proposed that various radicals could undergo addition to carbon–carbon double bonds to generate stable carbon-centered radicals, which could then be transformed through SET-mediated carbonylation. This approach is highly versatile, as numerous single-electron precursors and strategies are available for generating single-electron species. Therefore, we recognize that to develop reactions with the greatest potential impact, we need to extract from more diverse precursors, such as alkanes or alkyl halides.

**Figure 6:**
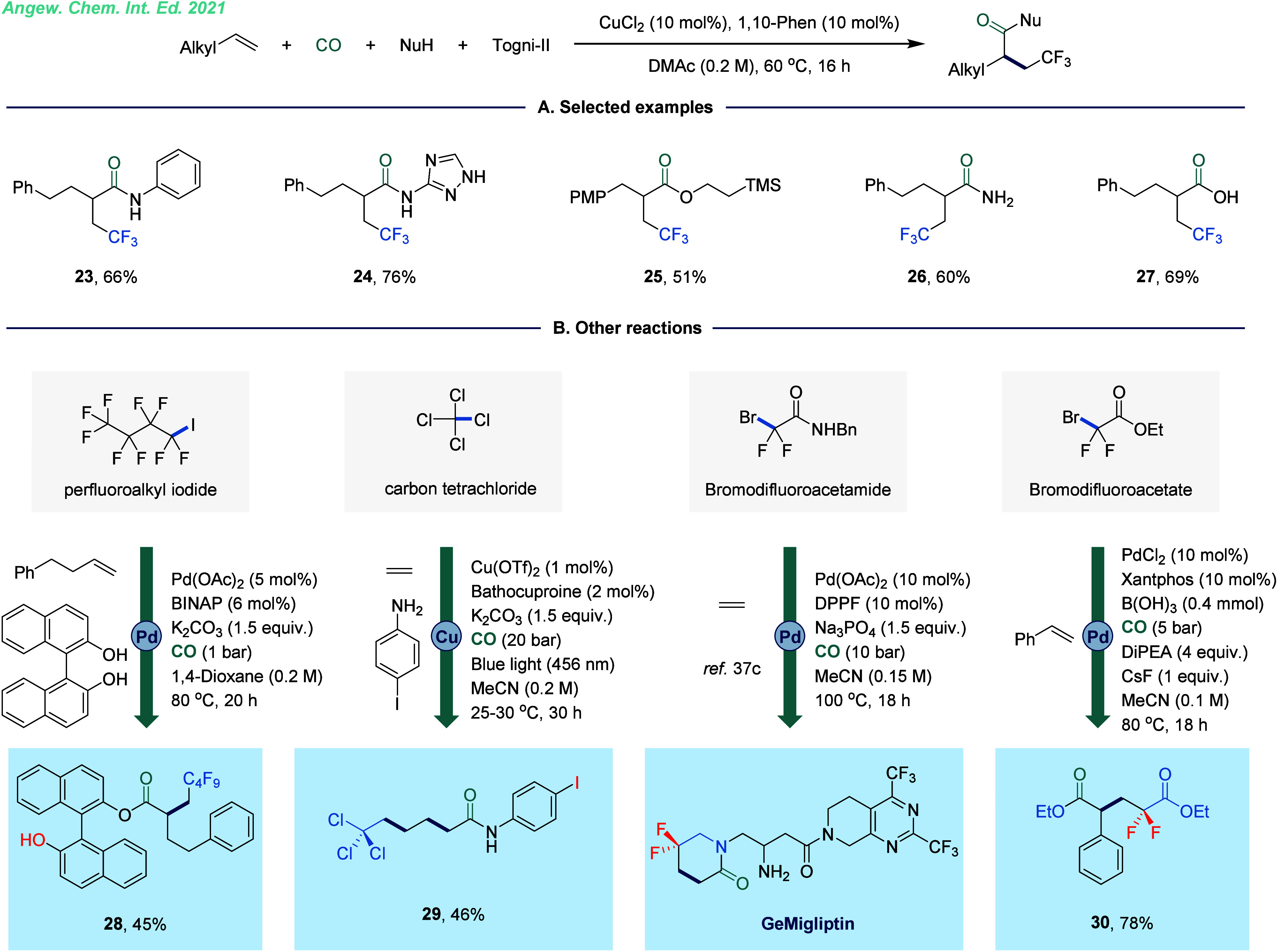
Copper-catalyzed 1,2-trifluoromethylation carbonylation of unactivated alkenes. A. Selected examples. B. Other examples for radical relay carbonylation of alkenes.

The trifluoromethyl (CF_3_) group holds significant potential in pharmaceuticals, agrochemical development, and materials science due to its unique physicochemical properties.^34^ In recent decades, substantial progress has been made in incorporating the CF_3_ group into organic molecules.^35^ Against this background, our group showcased a copper-catalyzed carbonylative trifluoromethylation of unactivated alkenes, including alkyl alkenes and ethylene, using Togni-II as an efficient trifluoromethyl source (Figure 6A).^36^ This transformation demonstrated remarkable efficiency, providing a straightforward method for concurrently introducing trifluoromethyl and carbonyl moieties. Moreover, the reaction proved applicable to a wide variety of amines and alcohols, enabling the generation of β-trifluoromethylated aliphatic carboxylic acid derivatives. Mechanistically, the reaction begins with the release of an electrophilic CF_3_ radical through a single-electron reduction process. Subsequently, the radical underwent addition chemistry with alkene to give a new carbon-centered radical, which was then trapped by the Cu(II) species to yield a Cu(III) intermediate. Following CO coordination and insertion, an acyl-copper intermediate is formed. This intermediate reacts with an amine to form the final product through reductive elimination, which regenerates the Cu(I) catalyst for the next catalytic cycle. Building on this work, we further investigated the SET-mediated radical relay carbonylation process (Figure 6B). This approach relies on precise control of reaction kinetics to ensure that each component participates sequentially and in an ordered manner.^37^ Using various electrophilic reagents and alkenes, the strategy enables the selective synthesis of a broad range of valuable carboxylic acid derivatives. By fine-tuning reaction conditions and optimizing the selection of electrophilic reagents, we can precisely control reaction pathways, facilitating efficient synthesis of the desired products and broadening the scope of applications in organic synthesis. This methodology not only enhances reaction selectivity and product diversity but also provides new strategies for synthesizing complex functionalized molecules.

**Figure 7:**
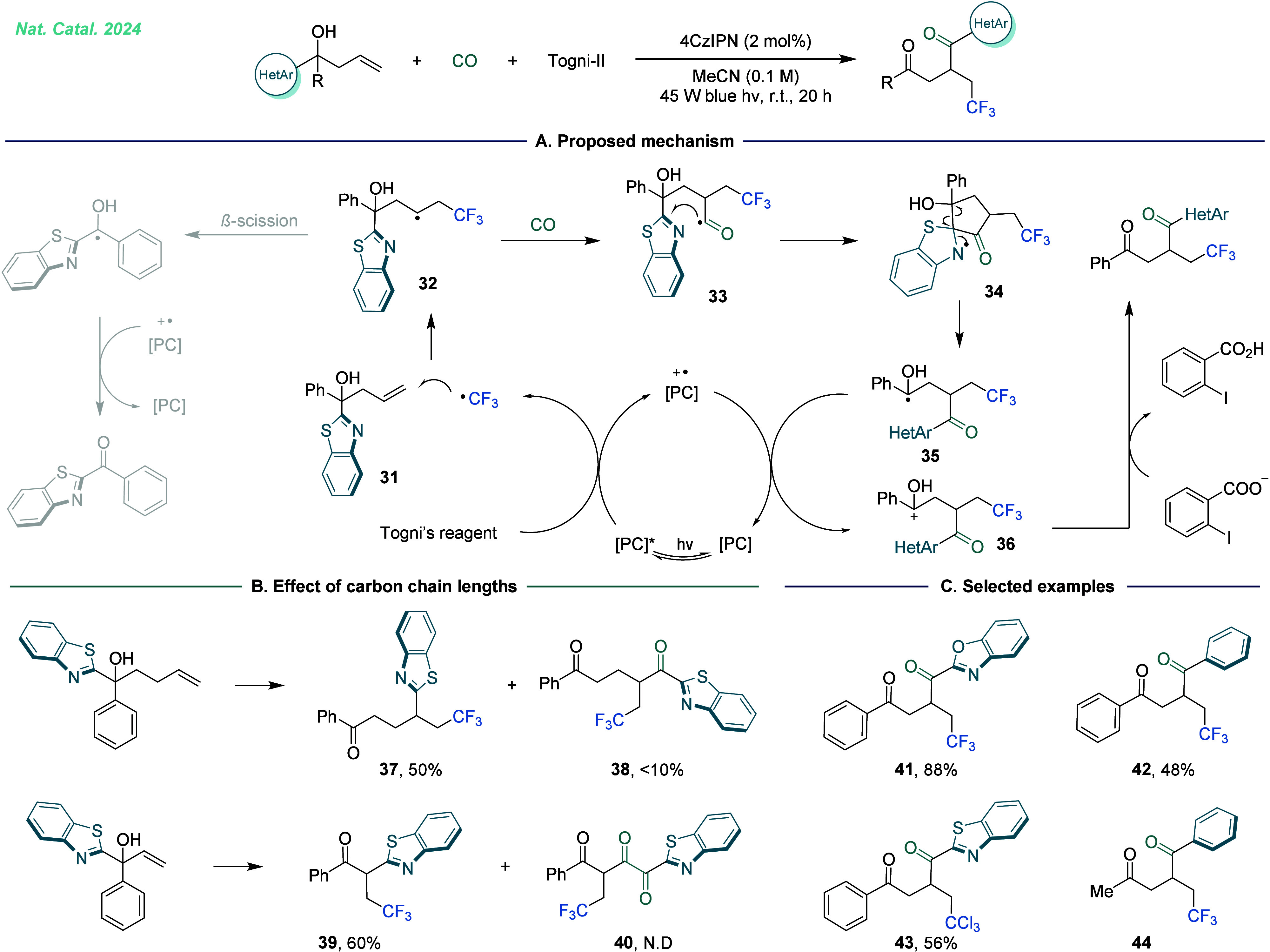
Carbon monoxide enabling synergistic carbonylation and (hetero)aryl migration. A. Proposed mechanism. B. Investigation on the reaction of substrate allylic alcohol and bishomoallylic alcohol. C. Selected examples. PC = Photocatalysts.

Carbon monoxide, as a simple and readily available C1 source, plays a crucial role in carbon chain extension reactions. Additionally, Additionally, CO serves as an essential precursor for many carboxylic acids and their derivatives, particularly in the production of key industrial chemicals.^38^ We aimed to further expand the irreplaceable roles of CO in organic synthesis. To this end, we unveiled a visible-light-induced radical relay rearrangement carbonylation process that operates in a nonclassical manner, with CO insertion serving as a pivotal step to facilitate functional group migration (Figure 7).^2^ In this transformation, the selective insertion of the carbonyl group into newly formed carbon radicals acts as a bridge for the migration of (hetero)aromatic groups. Notably, the positive feedback from functional group migration enhances the efficiency of carbon radicals in capturing CO, significantly improving the overall reaction efficiency. Mechanistically, the process began with the generation of a trifluoromethyl radical under visible light irradiation in the presence of photocatalysts. The radical then underwent addition to a carbon–carbon double bond, forming a new carbon-centered radical. The resulting carbon radical rapidly captured CO to generate acyl radical intermediate **33**. Due to the thermodynamic advantage of the five-membered cyclic transition state, intermediate **33** preferentially underwent an intramolecular cyclization to obtain the intermediate **34**. Aromatization then promoted the cleavage of the C–C bond, yielding a more stable α-OH carbon-centered radical intermediate **35**. Finally, the desired product is obtained through single-electron oxidation and subsequent deprotonation (Figure 7A). Although radical addition-functional group migration reactions of this nature have been extensively investigated, this process offered a compelling strategy for enabling the utilization of previously incompatible substrates in an alternative carbonylative (hetero)aryl migration reaction. This carbonylation-driven migration strategy not only assigns a novel role to CO but also injects momentum into rearrangement reactions and the migration of other functional groups facilitated by CO insertion. By harnessing the unique properties of CO, this methodology opens new pathways for functional group manipulation and expands the scope of carbonylation chemistry in organic synthesis.

**Figure 8:**
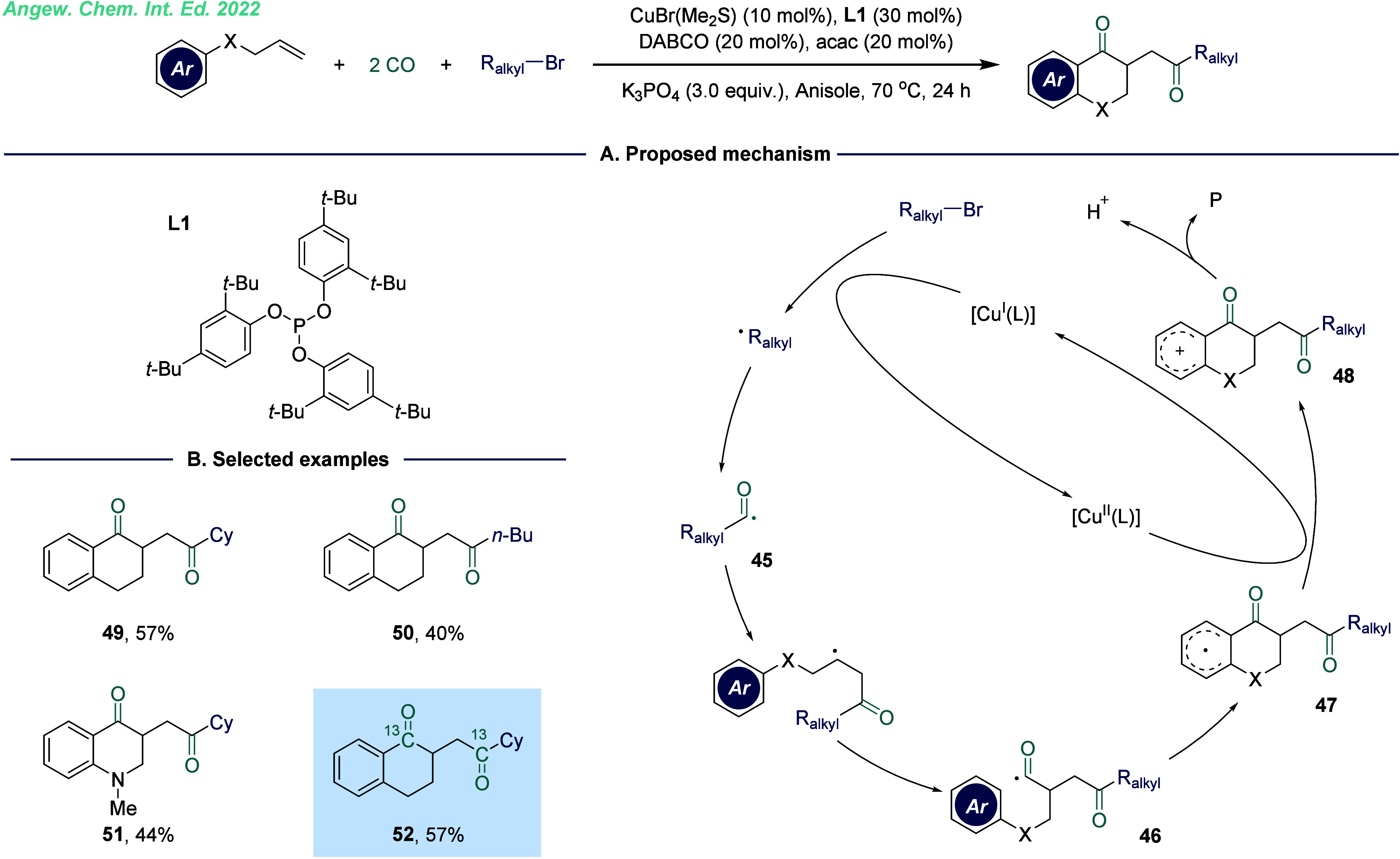
Copper-catalyzed 1,2-dicarbonylative cyclization of alkenes with alkyl bromides via radical cascade process. A. Proposed mechanism. B. Selected examples.

1,4-Diketones, as a critical structural backbone, have found widespread application in the field of organic synthesis.^39^ Building on our previous work, we envisioned that a controllable process could be developed by optimization reaction conditions, enabling selective synthesis of diketone derivatives in a four-component system involving alkyl halides, alkenes, and two equivalents of CO (Figure 8).^40^ Initially, the alkyl radical was generated via a single-electron-transfer process facilitated by Cu(I). This alkyl radical was then captured by CO, leading to the formation of the acyl radical intermediate **45**, incorporating the first CO molecule into the reaction. The acyl radical subsequently reacted with alkene, forming a new carbon radical. At this stage, the second CO molecule could be captured by the carbon radical, resulting in the generation of a second acyl radical intermediate **46** (Figure 8A). Through this pathway, the controllable and sequential insertion of two CO molecules was achieved. This reaction represents a highly efficient and versatile strategy, forming four new C–C bonds, two carbonyl groups, and one carbon-containing or heterocyclic ring in a single step. The ability to construct such complex molecular frameworks in a streamlined manner underscores the potential of this methodology for advancing the synthesis of functionalized diketone derivatives.

## 3 Single-Electron-Oxidation of C–H Bonds

**Figure 9:**
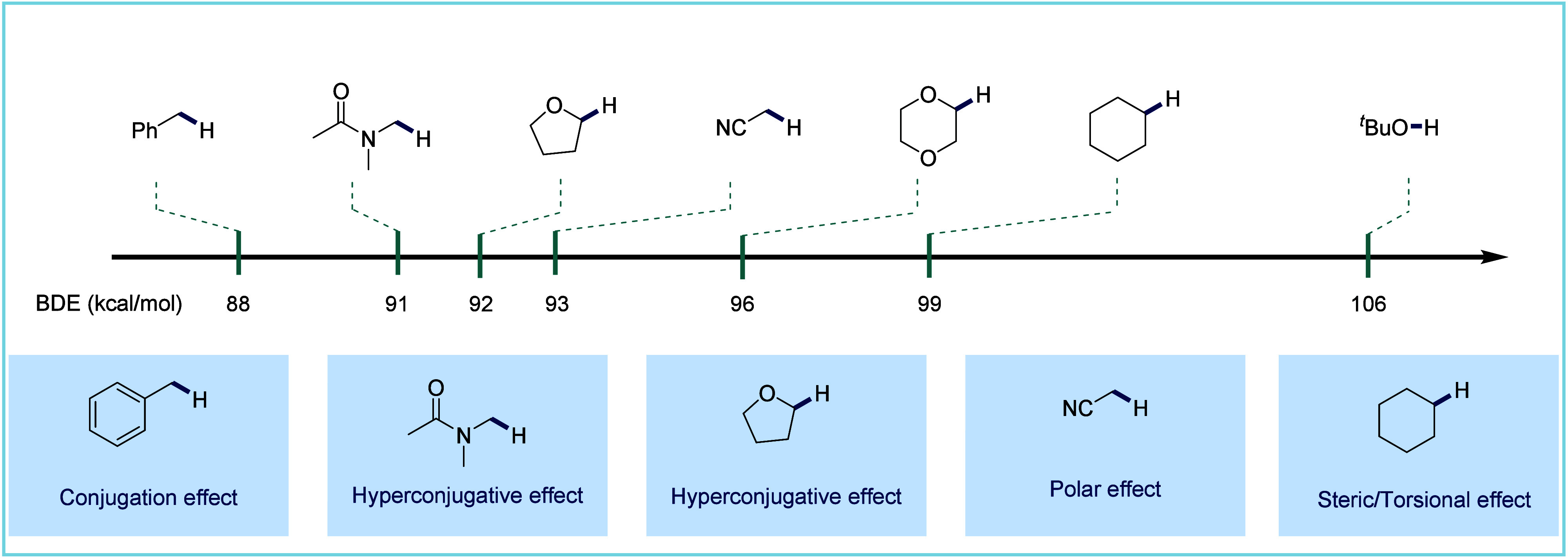
Bond dissociation energies (kcal mol^–1^) of C–H bonds in representative compounds. Possible factors affecting the properties of corresponding carbon radicals.

Although C–H bonds are ubiquitous in organic molecules, ranging from simple feedstock chemicals to complex natural products, their transformation presents a significant challenge.^41^ As Prof. Shilov and Prof. Shul’pin aptly stated, “to activate a σ-bond such as C–H bond is to increase the reactivity of this bond toward a reagent.^42^ This concept provides unique opportunities to use C–H bonds as atom-economic handles, enabling structural reconstruction and the rapid generation of molecular complexity. However, carbonylation reactions involving C(sp^3^)–H bonds remain challenging due to several factors: the thermodynamic barrier associated with cleaving inert C(sp^3^)–H bonds with high bond dissociation energies, the difficulty in selectively distinguishing between various C(sp^3^)–H bonds, and the reducing nature of the CO environment (Figure 9). Despite these challenges, advances in transition metal catalysis have led to the development of elegant catalytic systems. These systems often employ transition metals and chelation-assistance to enable various transformations.^43^ For C(sp^3^)–H bonds, we have found that in addition to the guiding group strategy, the hydrogen atom transfer (HAT) strategy can be employed in catalytic paradigms for efficient C–H carbonylation. Alternatively, CO can directly add to the carbon radical, generating an acyl radical. This process is facilitated under high-pressure CO conditions and with the assistance of transition metal catalysts.

**Figure 10:**
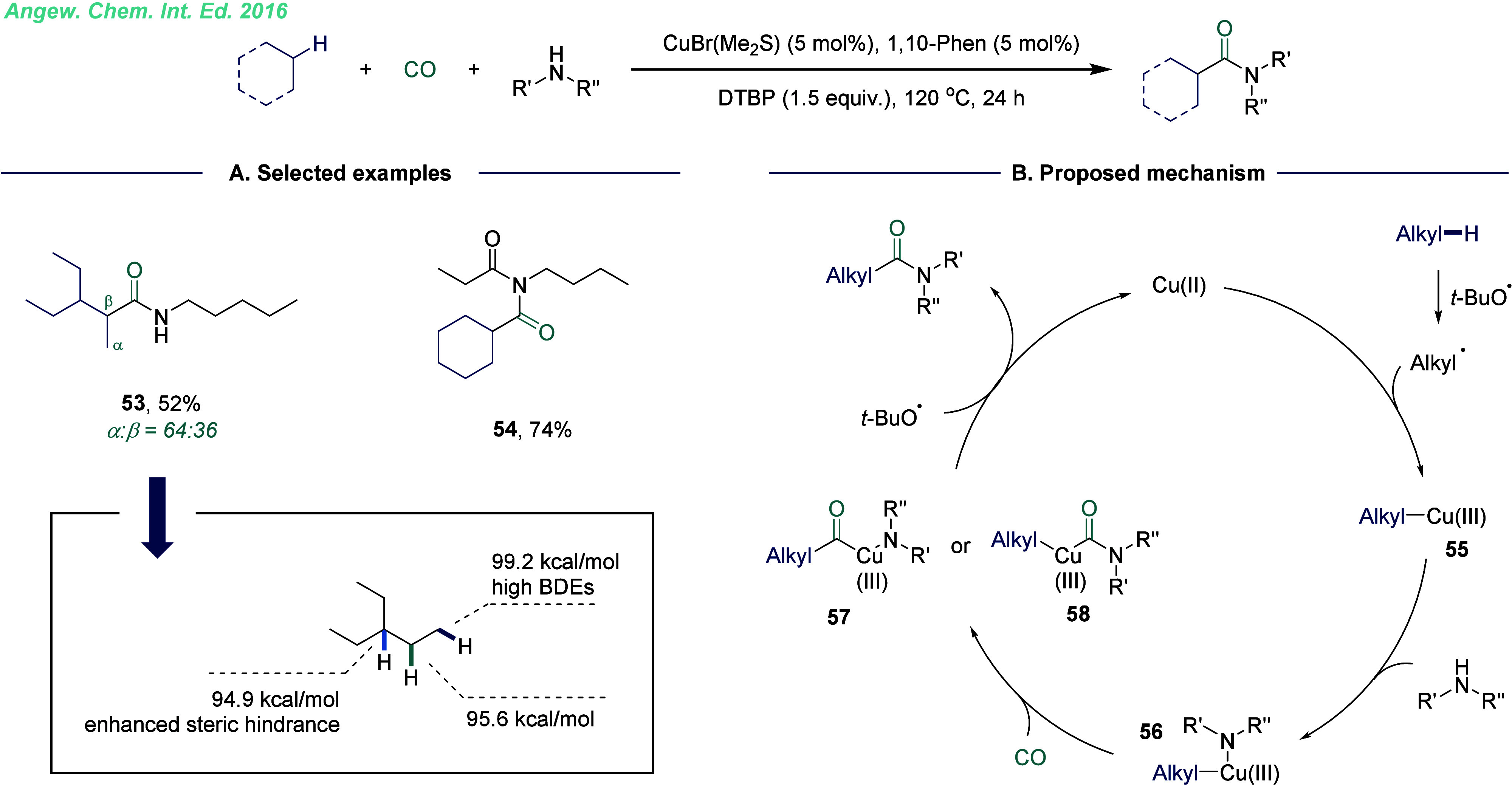
Copper-catalyzed carbonylative coupling of alkanes. A. Selected examples. B. Proposed mechanism. DTBP = Di-*tert*-butyl peroxide.

In 2016, our group developed the direct C–H bond cleavage and carbonylation strategy of alkanes using a catalytic loading of copper salts (Figure 10).^3,44^ This process enabled the efficient formation of imides and aliphatic amides in an atom- and step-economical manner. It demonstrated the broad applicability of the copper-catalyzed carbonylative platform for a diverse range of alkanes, including cycloalkanes and straight-chain alkanes (3-ethylpentane, pentane, and hexane), as well as adamantane. Additionally, weakly nucleophilic amides exhibited excellent reactivity in this transformation. Notably, this was the first reported example of copper-catalyzed carbonylative C–H activation. The reaction mechanism began with the thermal homolytic cleavage of di-*tert*-butyl peroxide (DTBP) and producing a *tert*-butoxy radical. This radical underwent a hydrogen atom transfer (HAT) process with the alkane, generating a carbon-centered radical. The carbon radical was then oxidized by Cu(I) to form a Cu(III)-alkane intermediate **55**. Intermediate **55** reacted with an amine to generate a Cu(III) intermediate **56**. Subsequent insertion of carbon monoxide produced intermediates **57** or **58**, which underwent reductive elimination to yield the final carbonylation product. The active Cu(I) catalyst was regenerated at the end of the catalytic cycle, ensuring the reaction’s efficiency and sustainability. The electronic push effect typically makes the tertiary carbon radical the most stable, which increases its tendency for decarbonylation. In contrast, the bond dissociation energies (BDEs) show that the primary carbon radical has the highest bond energy, making the activation of the C–H bond the least favorable.

**Figure 11:**
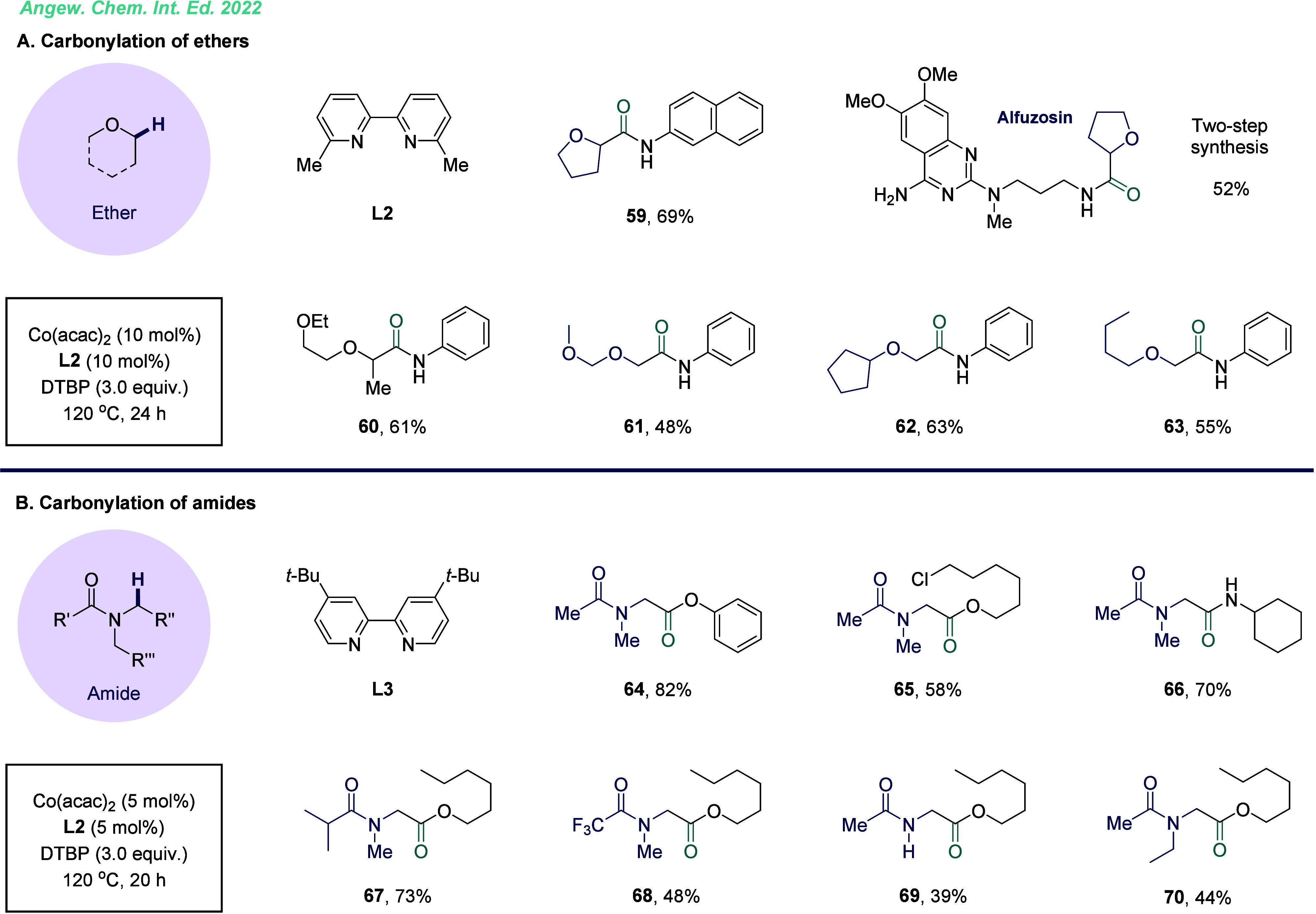
A. C–H carbonylative transformation of ethers. B. C–H carbonylative transformation of amides. acac = Acetylacetone.

The precise introduction of functional groups in heteroatom-containing compounds has garnered significant attention in the field of organic synthetic due to their prevalence in pharmaceuticals and functional materials.^45^ Simple ethers, widely present in biomass and various chemical feedstocks, serve as key starting materials for the production of value-added chemicals. The development of a general catalytic platform to convert these ethers into useful carboxylic acid derivatives would greatly enhance the rapid and convenient synthesis of a large number of bioactive molecules. Building on this concept, we reported a cobalt-catalyzed aminocarbonylation of ethers for the synthesis of α-amide substituted ether derivatives (Figure 11A).^46^ By employing unsymmetrical ethers, the process achieved terminal α-selective products (**61**–**63**) with excellent regioselectivity. Notably, Alfuzosin, a commercially available prescription medicine for treating high blood pressure, was prepared simply in this procedure with moderate yield.

Amides are one of the most common and versatile functional groups, playing vital roles in organic chemistry and medicinal chemistry. Approximately 40% of the 420 thousand bioactive molecules in the data set contain amides, and they constitute two-thirds of drug candidates. Furthermore, amide-containing drugs represent about 25% of all currently marketed pharmaceuticals.^47^ Developing a straightforward catalytic method for α-aminoalkyl carbonylation would provide an efficient route for synthesizing α-amino acids and their derivatives. However, activating α-aminoalkyl groups is particularly challenging due to the presence of easily oxidizable lone-pair electrons. In our research, we discovered that employing electron-withdrawing groups effectively reduces the nucleophilicity of α-carbon radicals while preventing excessive oxidation, thereby facilitating this transformation (Figure 11B).^48^

### 3.1 Oxidation-Induced Tandem Alkene Carbonylation

**Figure 12:**
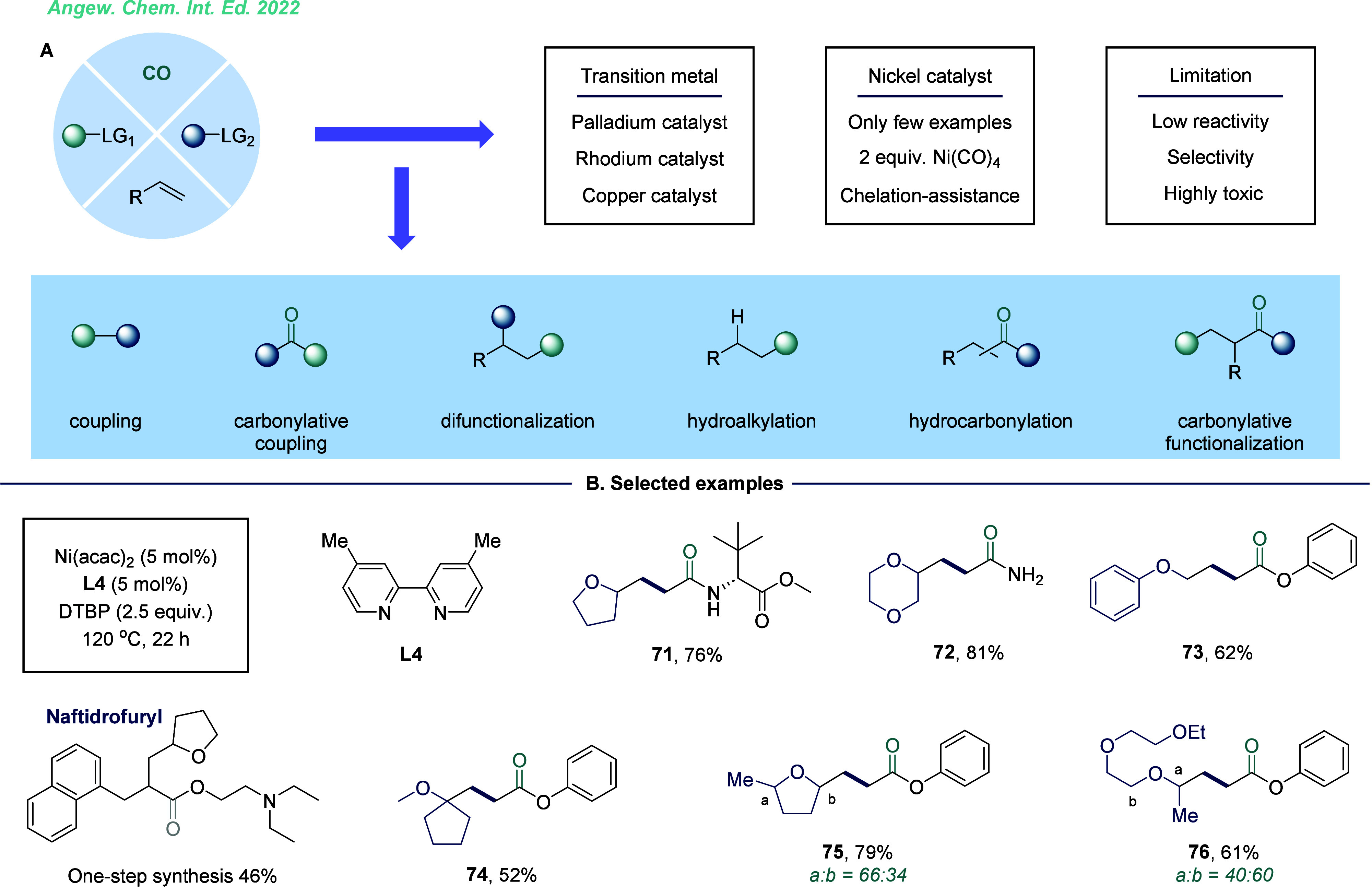
Nickel-catalyzed four-component carbonylation of ethers and alkenes. A. Challenges. B. Selected examples. LG = Leaving Group.

Carbonylative difunctionalization of alkenes is an efficient strategy for synthesizing carboxylic acid building blocks. Ethylene, the simplest olefin, plays a crucial role in the global chemical industry as a key industrial feedstock, with a global production of approximately 232 million tons projected for 2024.^49^ However, in terms of addition rate, electrophilic carbon radicals exhibit lower reactivity toward ethylene (∼10^3^ M^–1^ s^–1^) compared to substituted alkenes, making it part of the slow class of radical addition reactions.^50^ Additionally, ethylene and CO are prone to polymerization under high temperature and pressure, complicating selective transformations.^51^ Ensuring that each component assembles in the desired sequence within such systems places stringent demands on the catalytic platform. Inspired by our earlier work, we envisioned that in a multicomponent system containing ethers, alkenes, and CO, it would be feasible to selectively synthesize carboxylic acid derivatives by selecting appropriate catalysts. While palladium- and copper-catalyzed carbonylative difunctionalization of alkenes is well-established, nickel catalysis has received less attention, largely due to its propensity to form low-reactivity and highly toxic Ni(CO)*_n_* species. We found that oxidative conditions provide a promising pathway for nickel-catalyzed carbonylation. Recently, our group developed an oxidative-driven carbonylative difunctionalization of alkenes with ethers, using 1 bar CO as a carbonyl source (Figure 12B).^52^ This method demonstrated broad substrate applicability across various ethers, yielding γ-oxy carboxylic acid derivatives with good efficiency. The incorporation of ethene can generate new radical center for CO capture. However, due to the inherent nature of radical addition, the transformation exhibited poor selectivity for ethers containing α-C–H bonds in differing environments.

**Figure 13:**
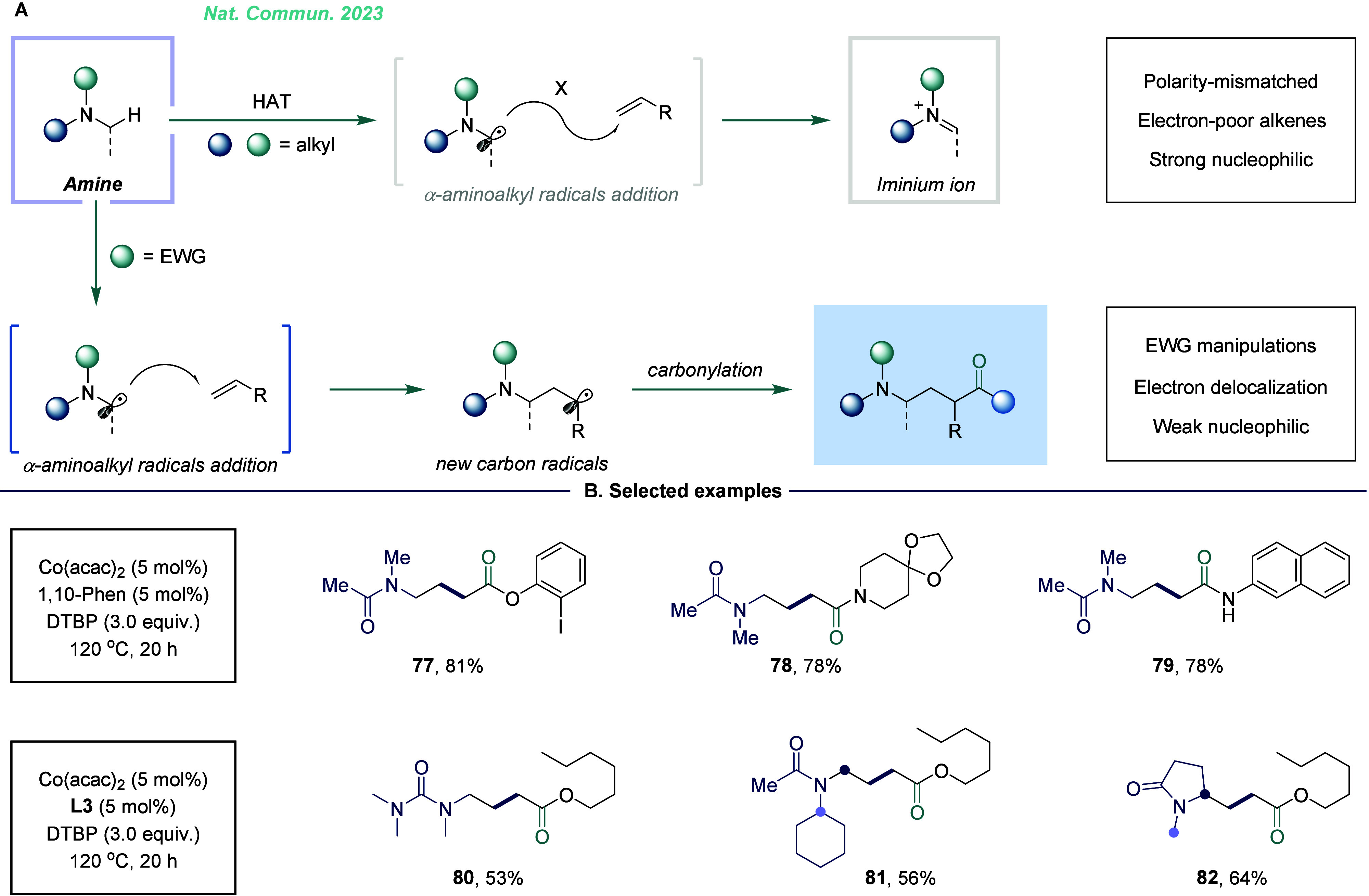
Aminoalkylative carbonylation of alkenes for the synthesis of γ-amino acid derivatives. A. Challenges and corresponding strategies. B. Selected examples.

The central goal of organic synthesis is to harness molecular diversity by assembling basic chemical building blocks. Among the various chemical motifs, amino acids play a pivotal role as essential components of proteins, pharmaceuticals, and biologically significant natural products.^53^ Specifically, γ-amino acids are widely used to modify peptide drugs, enhancing properties such as resistance to hydrolysis, half-life, pharmacokinetics, and physiological activity. Furthermore, as inhibitory neurotransmitters in the mammalian central nervous system (CNS), γ-amino acids have broad physiological functions, making them increasingly valuable in the medical and chemical industries.^54^ Through retrosynthetic analysis, alkenes, amines, and CO appear to be reasonable building blocks for the synthesis of γ-amino acids. However, compared to electrophilic carbon radicals, due to a polarity mismatch, α-aminoalkyl radicals exhibit lower reactivity toward unactivated alkenes, especially toward ethylene. In our research, we found that the use of an electron-withdrawing group effectively facilitated the addition reaction (Figure 13).^4^ These groups reduced the electron density of nitrogen, weakening its nucleophilicity and improving the reaction’s efficiency. This approach yielded good reactivity and high selectivity, particularly in transformations involving ethylene and multisite amides. This methodology highlights the potential of combining inexpensive catalysts with widely available starting materials to access valuable γ-amino acid derivatives. Additionally, we have successfully developed a copper-catalyzed carbonylative difunctionalization of acetonitrile, achieving high selectivity and moderate to good yields of the corresponding products.^55^ Building on this, we also designed a cost-effective cobalt-catalyzed carbonylative difunctionalization method for synthesizing γ-aryl carboxylic acid esters from readily available methylarenes, ethylene, and CO.^56^

## 4 Conclusions and Outlook

In this Account, we have summarized our recent advancements in SET-mediated carbonylation. Our research explored diverse carbonylation modes, expanding the scope and efficiency of these transformations. We first developed a copper-catalyzed system enabling selective mono- and double-carbonylation, as well as a phosphine-catalyzed, light-induced carbonylation of alkyl iodides with phenols under 1 bar of CO. For activated alkyl halides, a single-electron system was designed to achieve carbonylation while effectively suppressing dehydrohalogenation and nucleophilic coupling. Furthermore, various radical precursors were employed to perform radical relay carbonylation, producing a wide range of difunctionalized carbonyl-containing compounds under reduction conditions. To underscore the versatility of CO in organic transformations, we developed a carbonylation/(hetero)aryl migration procedure with CO as the trigger to prolong the carbon of the core structure. Subsequently, transition metal-catalyzed carbonylation of inert C(sp^3^)–H bonds was achieved, converting alkanes, ethers, amides, and other compounds into high-value carboxylic acid derivatives. Additionally, our group addressed the challenge of radical relay carbonylation of C–H bonds, a longstanding issue due to the high bond energy and electronic properties of the carbon radical. We successfully developed a multicomponent carbonylation method for unactivated alkenes starting from simple C(sp^3^)–H bonds. More importantly, our methodologies hold significant opportunities for pharmaceutical applications, as several commercially available drug molecules could be synthesized using our SET-mediated carbonylation approaches.

In our investigation of these transformations, we identified several unresolved challenges in this domain. While we emphasize the successful implementation of various catalytic modes to achieve the binding of CO with chemicals having different reduction or oxidation potentials. However, the mechanistic ambiguity regarding whether the generated radical is first captured by CO or by the metal catalyst. This uncertainty complicates mechanistic studies and can pose challenges for researchers new to the field. Since it is difficult to rule out either possibility, in most cases, we tend to consider both possibilities simultaneously. Additionally, alcohols are readily available, structurally diverse, and operationally convenient native alkyl building blocks. Utilizing alcohols as catalytic handles could significantly advance SET-mediated carbonylation. However, the low electrophilicity and innate strength of the C(sp^3^)–O bonds pose substantial challenges for catalytic carbonylation. Moreover, in C(sp^3^)–H carbonylation, continuous efforts need to be dedicated to innovating new catalytic systems based on selective and mild hydrogen transfer reagents. Addressing the issue of single-selectivity carbonylation of C(sp^3^)–H bonds and promoting green, mild reaction conditions are also crucial goals. To overcome these challenges, innovative strategies are needed, including the design of new catalysts or photometallic chemical synergistic strategies. These approaches hold promise for efficiently activating inert chemical bonds in the presence of CO. Our research group is actively pursuing these challenging reactions, aiming to further expand the potential of SET-mediated carbonylation and contribute to the development of sustainable and efficient catalytic systems.
